# Experience developing national evidence-based clinical guidelines for childhood pneumonia in a low-income setting - making the GRADE?

**DOI:** 10.1186/1471-2431-12-1

**Published:** 2012-01-01

**Authors:** Ambrose Agweyu, Newton Opiyo, Mike English

**Affiliations:** 1 Kenya Medical Research Institute - Wellcome Trust Research Programme, Nairobi, Kenya; 2 Department of Paediatrics, University of Oxford, Oxford, UK

## Abstract

**Background:**

The development of evidence-based clinical practice guidelines has gained wide acceptance in high-income countries and reputable international organizations. Whereas this approach may be a desirable standard, challenges remain in low-income settings with limited capacity and resources for evidence synthesis and guideline development. We present our experience using the Grading of Recommendations Assessment, Development and Evaluation (GRADE) approach for the recent revision of the Kenyan pediatric clinical guidelines focusing on antibiotic treatment of pneumonia.

**Methods:**

A team of health professionals, many with minimal prior experience conducting systematic reviews, carried out evidence synthesis for structured clinical questions. Summaries were compiled and distributed to a panel of clinicians, academicians and policy-makers to generate recommendations based on best available research evidence and locally-relevant contextual factors.

**Results:**

We reviewed six eligible articles on non-severe and 13 on severe/very severe pneumonia. Moderate quality evidence suggesting similar clinical outcomes comparing amoxicillin and cotrimoxazole for non-severe pneumonia received a strong recommendation against adopting amoxicillin. The panel voted strongly against amoxicillin for severe pneumonia over benzyl penicillin despite moderate quality evidence suggesting clinical equivalence between the two and additional factors favoring amoxicillin. Very low quality evidence suggesting ceftriaxone was as effective as the standard benzyl penicillin plus gentamicin for very severe pneumonia received a strong recommendation supporting the standard treatment.

**Conclusions:**

Although this exercise may have fallen short of the rigorous requirements recommended by the developers of GRADE, it was arguably an improvement on previous attempts at guideline development in low-income countries and offers valuable lessons for future similar exercises where resources and locally-generated evidence are scarce.

## 

The approach to developing clinical practice guidelines has become increasingly formal over the last two decades. Requirements now span rigor in evidence appraisal to incorporation of user preferences. As a result developed countries have invested substantially in national institutes to develop guidelines (National Institute for Health and Clinical Excellence [1],; Agency for Healthcare Research and Quality [2], Norwegian Knowledge Centre for the Health Services [3] etc). The World Health Organization (WHO), after criticisms of its guideline development procedures [4], recently adopted the Grading of Recommendations Assessment, Development and Evaluation (GRADE) approach [5] expecting it to be used for all new technical advice [6]. Some of the challenges of using GRADE or similar structured approaches to guideline development at international and national levels in developed economies are emerging [7,8]. However, reports documenting experiences from low-income settings are lacking. Yet the GRADE approach, acknowledging that evidence alone is inadequate for making recommendations, specifically directs that local contextual factors be taken into account when producing recommendations. This has resulted in more transparently developed guidance than in the past, where guideline development usually took the form of a small meeting of experts behind closed doors. However the price of this has been increasingly formal procedures making new demands on limited national capacity.

In 2009 the Kenyan Ministry of Medical Services requested support to revise the national pediatric guidelines. We decided, with limited technical and financial resources, to attempt the use of the GRADE approach (see summary in Table 2) for this national exercise. Here we illustrate, from the perspective of those reviewing, summarizing and presenting the evidence in a resource-constrained setting, challenges encountered during this process and when moving from evidence to recommendations. In doing this we seek to go beyond the current focus on further improvements in methodology [9] and broaden the discussion to questions around implementation of GRADE procedures in low income settings. Although we attempted to tackle eleven guideline-related questions we focus here, as an exemplar, on questions identified when attempting to update the case-management guideline for pneumonia in children.

### Background to childhood pneumonia guidelines

Childhood pneumonia continues to rank as the leading cause of hospitalization and death in children globally [10]. The current Kenyan guidelines (Table 1) for antibiotic treatment of community-acquired childhood pneumonia are adapted from those of WHO and recommend classification of children into one of 3 clinical categories of severity to guide decisions on appropriate treatment [11]. Key treatment recommendations for children without HIV infection are largely unchanged since their first launch over twenty years ago and concerns have been expressed over their current and future appropriateness [12]. Perhaps linked to such concerns there is evidence of poor guideline adherence revealing possible preferences for 'stronger' (broader spectrum, non-beta-lactam) antibiotics [13].

**Table 1:** 2005 GoK clinical classification and recommended antibiotic treatment of children with cough and/or difficulty breathing

Syndrome	Clinical criteria	Recommended antibiotic treatment
**Non-severe pneumonia**	Rapid breathing: (≥ 50 if 2 - 11 months) (≥ 40 if 12 - 59 months)	Out patient with oral cotrimoxazole

**Severe pneumonia**	lower chest indrawing and HIV-unexposed	Inpatient treatment with IV/IM benzyl penicillin monotherapy)

**Very severe pneumonia**	Unable to drink, central cyanosis, altered level of consciousness, head nodding, grunting and HIV-exposed	Inpatient treatment with IV/IM benzyl penicillin) and gentamicin combination therapy OR IV/IM chloramphenicol) (plus high dose cotrimoxazole, 8 mg/kg trimethoprim and 40 mg/kg of sulfamethoxazole 6 hourly I.V/oral if HIV-exposed)

GoK - Government of Kenya

### GRADE, GRADE-lite or an inability to make the GRADE?

When resources are limited compromises are made. We illustrate such compromises here both to indicate where sharing resources, capacity and prior work may be helpful and because they raise the question of whether or not what we describe, as an illustration of what may be possible in low income settings, is a 'GRADE-compliant' process.

### Defining the clinical questions and relevant outcomes

Our task was updating of pediatric national guidelines in a period of 9 months. In a recent report an international guideline development group engaged an extensive network of experts taking into account multidisciplinary expertise, and regional and gender representation to determine policy-relevant questions [14]. Such an elaborate process was not possible in our case. Policy relevant questions were thus based on the scope of prior guidelines, working knowledge of topic areas and observed local clinical practice, discussions with a small number of key-informants in government and *a priori *considerations of what might be feasible. Similarly, we defined within the 'GRADE-team' predominantly critical outcomes (mortality and morbidity) that spanned all guideline topics under review. This approach was used to help standardize procedures and in anticipation of presentation and discussion with a national recommendations panel likely to have limited experience considering research evidence. PICO (Population, Intervention, Comparator, Outcome) formatted questions (Table 2) were thus constrained from the outset by a modest set of opinions from those within the GRADE-team and local observations. This was in large part driven by limited resources and an absence of mechanisms to rapidly gain wider opinions from key sources including patients, caregivers and policy makers. Although this relatively narrow focus has the potential for introducing bias such compromises seem inevitable in the short to medium term in settings like Kenya.

**Table 2:** Summary of the GRADE system for guideline development

We used the GRADE system to develop the recommendations since it provides an explicit and transparent assessment of the quality of evidence and the strength of recommendations for clinical questions.			
PICO, an acronym that represents four attributes (Population, Intervention, Comparator and Outcome) is widely used in formulation of clinical questions. The PICO elements for our 5 clinical questions of interest for childhood pneumonia in the Kenyan treatment guidelines are given below.			

**Population: *Kenyan children aged 2 - 59 months meeting WHO criteria for*:**	**Intervention (proposed new treatment)**	**Comparator (standard treatment)**	**Outcomes of interest**

***i. non-severe pneumonia ***	I. Amoxicillin	Co-trimoxazole	i. Mortality
	
***ii. severe pneumonia. (HIV-unexposed without severe malnutrition) ***	II. Amoxicillin	Benzyl penicillin/ampicillin	ii. Treatment failure
		
	III. Benzyl penicillin/ampicillin plus gentamicin	Benzyl penicillin (or amoxicillin)	OR
	
***iii. very severe pneumonia. (HIV-unexposed without severe malnutrition) ***	IV. Benzyl penicillin/ampicillin plus gentamicin	Chloramphenicol	Time to resolution of signs of pneumonia
		
	V. Ceftriaxone	Benzyl penicillin/ampicillin plus gentamicin	iii. Cost

GRADE classifies quality of evidence into four categories (high, moderate, low, or very low) and recommendations (for or against treatments) into two grades (strong or weak). With study design as the starting point, RCTs ranking highest and observational studies lowest, it allows for downgrading of the quality of evidence in the presence of factor related to study limitations, consistency, directness and publication bias. It also allows for upgrading the quality of evidence in the presence of large treatment effects, a dose-response gradient and residual confounding likely to underestimate the true effect. The current revised interpretation of the GRADE levels of evidence is shown below.			

**Quality level**	**Interpretation**		

**High**	Highly confident that the true effect lies close to that of the estimate of the effect		

**Moderate**	Moderately confident in the effect estimate: The true effect is likely to be close to the estimate of the effect, but there is a possibility that it is substantially different		

**Low**	Limited confidence in the effect estimate: The true effect may be substantially different from the estimate of the effect		

**Very low**	Very little confidence in the effect estimate: The true effect is likely to be substantially different from the estimate of effect		

Unlike other guideline development tools, GRADE uniquely separates judgments on the quality of evidence and the strength of recommendations - recognizing that making recommendations involves tradeoffs between benefits and harms, and contextual factors (e.g. costs, baseline risk of population, clinician values and preferences, etc). Thus, using the above assessments, we classified recommendations into categories summarized in figure 2.			

We defined the following clinical questions regarding antibiotic treatment for children aged 2 - 59 months with non-severe, severe and very severe pneumonia:

1. For children with non-severe pneumonia, should cotrimoxazole be replaced by amoxicillin?

2. For children with severe pneumonia, should benzyl penicillin be replaced by oral amoxicillin?

3. For children with severe pneumonia, should benzyl penicillin monotherapy be replaced by benzyl penicillin plus gentamicin?

4. For children with very severe pneumonia, should chloramphenicol be abandoned as an alternative treatment to benzyl penicillin plus gentamicin?

5. For children with very severe pneumonia, should benzyl penicillin plus gentamicin be replaced by ceftriaxone?

Children exposed to, or infected with HIV and those with severe acute malnutrition were excluded from our population of interest due to the unique treatment considerations among these groups.

### Bases for the clinical questions

The decision to review clinical questions 1 and 4 was based on recent WHO technical updates. These recommend the use of amoxicillin in favor of cotrimoxazole in regions with high resistance to cotrimoxazole and benzyl penicillin plus gentamicin in preference to chloramphenicol for very severe pneumonia [15]. Evidence from Asian studies suggesting comparable effectiveness of oral amoxicillin and benzyl-penicillin for treatment of severe pneumonia led us to review clinical question 2. Widespread use of benzyl penicillin plus gentamicin and ceftriaxone among Kenyan clinicians for the treatment of severe and very severe pneumonia respectively, contrary to the recommended guidelines [13], prompted our choice of clinical questions 3 and 5.

### Evidence retrieval, assessment and synthesis

Our approach to literature searching and summary are outlined in Table 3 and Figure 1. The technical and human resource capacity for accessing literature and for undertaking systematic reviews in low-income country settings, while slowly improving, remains limited. In an effort to ensure a participatory process and despite the absence of 'professional' guideline developers, we engaged government pediatricians in the review process. This meant for all topics, including pneumonia, that only 2 reviewers independently appraised available literature, reaching consensus by discussion where required. In some cases reviewer pairs had very similar professional backgrounds and very limited experience in literature appraisal although limited training and access to technical support were provided over the 8 months preparation phase (Figure 2). However, further investment in quality assurance of the review process was beyond the resources and capacity of the group, making errors or misjudgments possible, perhaps particularly when examining topics without existing well conducted systematic reviews. Despite our focus on the major killers of children including pneumonia, malaria, neonatal sepsis and malnutrition, absence of systematic reviews was common and where reviews were available none had GRADE summary of evidence tables (with searches up to March 2010).

**Table 3:** Search strategy for clinical questions for pneumonia

We searched PubMed and the Cochrane Library. Both free text and MeSH terms were used in the PubMed search as shown below.

Search strategy: pneumonia AND (child* OR paediatric OR pediatric) AND (antibiotic OR co-trimoxazole OR cotrimoxazole OR sulfamethoxazole OR sulphamethoxazole OR penicillin OR amoxicillin OR amoxycillin OR cephalosporin OR ceftriaxone OR gentamicin OR chloramphenicol OR quinolone* OR levofloxacin OR macrolide* OR erythromycin).

Systematic reviews and randomized controlled trials relevant our questions of interest were reviewed. Where none were available, observational studies were considered. The search was not limited by date or language. In addition, bibliographies of eligible articles were screened for additional reports warranting inclusion. Abstracts for all retrieved articles were read and the full text articles for those addressing the policy question of interest selected. Searches around the 3 broad policy questions were conducted in February 2010 and the results reviewed independently by A.A. and M.E. We then summarized the evidence from the retrieved literature for each policy question in the form of mini-reviews and key finding summaries (all available at http://www.idoc-africa.org).

**Figure 1:**
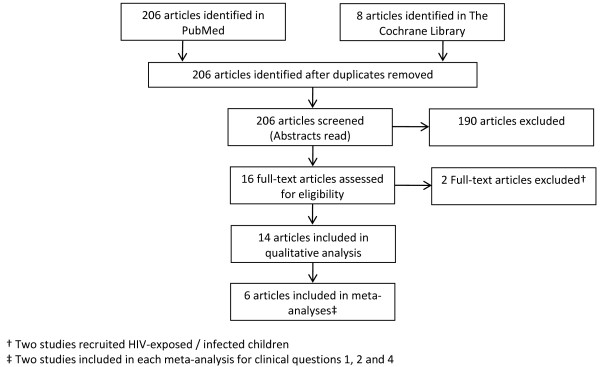
**Flow diagram of search strategy used for selecting studies for review**.

**Figure 2:**
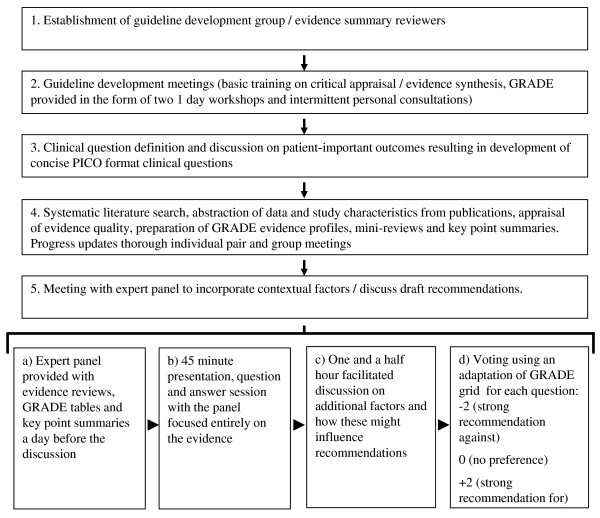
**Steps in the development of the revised 2010 Kenyan pediatric treatment guidelines**.

### Grading the quality of evidence

Of the 14 team members involved in the entire process only 2 (ME and NO) had experience conducting systematic reviews and limited, prior exposure to the GRADE approach. However, supported by one key team member (NO), software developed by the GRADE Working Group was utilized to classify the quality of available evidence into four categories (Table 2) after upgrading and downgrading based on our perceptions of merits and weaknesses respectively. As has been pointed out elsewhere [7] there are no absolute criteria for up and down-grading decisions and while GRADE helps make the process explicit the decisions remain to a degree subjective. Where possible, we conducted meta-analyses to improve the precision around estimated effects using RevMan version 5 (Cochrane Collaboration) and subsequently presented the combined data in the GRADEpro software [16]. The meta-analyses included all randomized controlled trials reporting common outcomes and excluded cluster randomized controlled trials.

We now illustrate some of the challenges we found in making these decisions in the case of pneumonia. Before doing this we provide a very concise summary of the primary evidence for selected questions with their GRADE quality of evidence tables. Our full evidence summary, that is entirely consistent with a recently published systematic review (that does not include GRADE tables) [17] can be accessed online [18].

### The evidence

We searched online databases, PubMed and The Cochrane Library using a common search strategy displayed in Table 3 to identify randomized controlled trials (RCTs) relevant to our questions of interest. The Therapy category and Narrow scope options under the Clinical Queries filter were applied within PubMed. Figure 1 illustrates the process used to select the studies we considered for review.

#### Non-severe pneumonia

For the policy question "should cotrimoxazole be replaced by amoxicillin in Kenyan children aged 2 - 59 months fulfilling the WHO criteria for non-severe pneumonia?" our search (see Table 3) retrieved 30 publications. Of the 6 eligible articles shortlisted, three were reviews, Kabra et al 2006 (Cochrane) [19] - updated in 2010 [17], Ayieko et al 2007 [20] and Grant et al 2009 [21] and three randomized controlled trials [22-24]. None of the trial data reported mortality as a primary outcome. The estimated effect of the intervention on treatment failure in all three trials was similar to that among children receiving the standard treatment. The quality of evidence from all three studies was downgraded for indirectness with respect to the populations studied since all were conducted in Asia (two in Pakistan [22,23] and one in India [24]). No serious limitations, inconsistencies or imprecision were identified in the studies reviewed, resulting in a conclusion of moderate quality evidence suggesting no difference between the two treatments. Our GRADE evidence summary for these studies is presented in Table 4.

**Table 4:** GRADE evidence profile 1: Cotrimoxazole versus amoxicillin for non-severe pneumonia^†^

Quality assessment					Summary of findings			
					**Number of patients**		**Effect**	**Quality of evidence**

**No of studies/Design**	**Limitations**	**Inconsistency**	**Indirectness**	**Imprecision**	**Amoxicillin**	**Cotrimoxazole**	**Odds ratio** **(95% CI)**	

*Outcome 1: Treatment failure based on clinical signs. Follow-up 2 - 5 days*. *Importance: Critical*								

2 RCTs [22,23]	no serious limitations	no serious inconsistency	serious^1^	no serious imprecision^3^	147/922 (15.9%)	231/1132 (20.4%)	0.83 (0.65 - 1.07)	Moderate

*Outcome 2: Treatment failure based on clinical signs. Follow-up 4-6 days*. *Importance: Critical*								

1 cluster randomised trial [24]	no serious limitations	no serious inconsistency	serious^2^	no serious imprecision^3^	137/993 (13.8%)	97/1016 (9.5%)	1.52 (0.73 - 3.17)	Moderate

*Outcome 3: Mortality* *Importance: Critical*								

No studies	-	-	-	-	-	-	-	-

*Outcome 4: Cost* *Importance: Important*								

No studies	-	-	-	-	-	-	-	-

**Overall quality of evidence: Moderate quality evidence suggests no difference between standard and proposed treatments for outcomes assessed**								

Benefits or desired effects:	Amoxicillin may be also effective for treatment of severe pneumonia, potentially simplifying treatment by reducing severity classes to two.							

Risks or undesired effects	Potential for increased bacterial resistance to amoxicillin with widespread use. Reduced options for second line treatment in case of treatment failure - a whole new class of antibiotic might have to be provided as second line treatment.							

Values and preferences:	Cotrimoxazole is formulated as a commonly used tablet in adults too that can be divided for children if pediatric formulations are missing - the same is not true for amoxicillin that is often distributed as capsules if syrups are not available							

Costs:	Amoxicillin is more costly than cotrimoxazole (US$ 0.12 and US$ 0.21 for cotrimoxazole and amoxicillin syrups respectively for a course appropriate for a child weighing approximately 10 kg [KEMSA* July 2009])							

Feasibility	Both drugs are widely available and in use							

^† ^Clinical question: For Kenyan children aged 2 - 59 months who meet WHO criteria for non-severe pneumonia, should cotrimoxazole be replaced by amoxicillin?

^1 ^Indirectness of population (studies conducted in Pakistan)

^2 ^Indirectness of population (study conducted in India)

^3 ^Although studies failed to show a difference between the two treatments, narrow confidence intervals and power calculation described by authors supported decision against downgrading the quality for imprecision.

KEMSA - Kenya Medical Supplies Agency

#### Severe pneumonia

Among children with severe pneumonia we sought to address the following questions:

1) Should injectable benzyl penicillin be replaced by oral amoxicillin?

2) Should injectable benzyl penicillin monotherapy be replaced by benzyl penicillin plus gentamicin?

We identified 8 articles including 2 Cochrane systematic reviews that addressed the two questions.

#### (a) Antibiotic treatment of severe pneumonia: benzyl penicillin/ampicillin versus amoxicillin

Three trials compared oral versus parenteral treatment for severe pneumonia: Addo-Yobo et al [17,20,25,26] conducted a large multi-centre trial of 1702 children in Colombia, Ghana, India, Mexico, Pakistan, South Africa (two sites), Vietnam, and Zambia while Atkinson et al [17,26,27] recruited 203 children with radiologically-confirmed community acquired pneumonia in the UK and Hazir et al [17,28] studied 2100 Pakistani children. All three trials, supported by a meta-analysis of the results of the studies by Hazir et al and Addo-Yobo et al which showed a pooled risk ratio of treatment failure for the two studies of 0.97, 95% CI 0.83 - 1.14, suggested equivalence of the two treatments. Since the studies were conducted among predominantly non-African populations the quality of evidence was downgraded by one level for indirectness. The evidence was therefore graded as moderate quality suggesting equivalence comparing benzyl penicillin and amoxicillin (Table 5).

**Table 5:** GRADE evidence profile 2: Benzyl penicillin versus amoxicillin for severe pneumonia^†^

Quality assessment					Summary of findings			
					**Number of patients**		**Effect**	**Quality of evidence**

**No of studies/Design**	**Limitations**	**Inconsistency**	**Indirectness**	**Imprecision**	**amoxicillin**	**benzyl penicillin**	**Odds ratio** **(95% CI)/P value**	

*Outcome 1: Treatment failure based on clinical signs. Assessed at 48 hours *[25]*and 5 days *[28] *Importance: Critical*								

2 RCTs [25,28]	no serious limitations	no serious inconsistency	serious^1^	no serious imprecision	244/1882 (13.0%)	248/1857 (13.4%)	0.97 (0.80-1.17)	Moderate

*Outcome 2: Time to resolution of signs of pneumonia* *Importance: Critical*								

1 RCT [27]	no serious limitations	no serious inconsistency	serious^2^	no serious imprecision	100 (1.3 days) ^†^	103 (1.3 days)^†^	P = 0.001 for equivalence	Moderate

*Outcome 3: Mortality* *Importance: Critical*								

No studies	-	-	-	-	-	-	-	-

*Outcome 4: Cost* *Importance: Important*								

No studies	-	-	-	-	-	-	-	-

**Overall quality of evidence: Moderate quality evidence suggests that the two treatments are equivalent for outcomes assessed**								

Benefits or desired effects	Safety of oral over injectable treatments, convenient dosing schedule (twice daily for amoxicillin versus four times a day for benzyl penicillin)							

Risks or undesired effects	None identified							

Values and preferences	Painless oral administration for amoxicillin preferable to injectable route required for benzyl penicillin/ampicillin, Mothers like injections; Mothers would not stay in hospital for oral medications; Staff would not feel they were giving a strong enough treatment for a severe disease							

Costs	Potential reduction in cost of resources required for injectable treatment including the option of out-patient management							

Feasibility	Both antibiotics widely available and in use							

^† ^Clinical question: For HIV-unexposed Kenyan children aged 2 - 59 months without clinical signs of severe malnutrition who meet WHO criteria for severe pneumonia, should parenteral benzyl penicillin/ampicillin be replaced by inpatient oral amoxicillin?

^1 ^Indirectness of population (one study conducted in Pakistan, one multicentre, multi-country)

^2 ^Indirectness of population (study conducted in the UK among children with radiologically confirmed pneumonia)

**^†^**Median duration to resolution of signs of pneumonia

#### (b) Antibiotic treatment of severe pneumonia: benzyl penicillin/ampicillin monotherapy versus benzyl penicillin/ampicillin and gentamicin

Only one small trial was found comparing costs and clinical outcomes enrolling 40 children with severe pneumonia in Malaysia in 1999/2000 after randomization to either benzyl penicillin/ampicillin monotherapy or combination therapy with gentamicin [17,29]. The results of this trial showed no differences in clinical outcome between the two treatments and higher costs associated with the combination of ampicillin and gentamicin.

#### Very severe pneumonia

Two policy questions were addressed relating to antibiotic treatment of children with very severe pneumonia:

1) Should chloramphenicol be abandoned as an alternative treatment to benzyl penicillin plus gentamicin?

2) Should benzyl penicillin plus gentamicin/chloramphenicol be replaced by ceftriaxone?

Two trials reviewed addressed each of the two questions. The studies were also summarized in a Cochrane review [17].

#### (c) Antibiotic treatment of very severe pneumonia: chloramphenicol versus benzyl penicillin/ampicillin and gentamicin

Two trials compared the effectiveness of benzyl penicillin/ampicillin combined with gentamicin versus chloramphenicol for very severe pneumonia. Duke et al (2002) studied 1116 children in Papua New Guinea [17,20,30] while Asghar et al (2008) recruited 958 children from eight sites in seven developing countries [17,31]. A meta-analysis of the two studies yielded a pooled risk ratio of treatment failure of 0.79, 95% CI 0.66 - 0.94 in favor of penicillin/ampicillin plus gentamicin.

#### (d) Antibiotic treatment of very severe pneumonia: benzyl penicillin/ampicillin and gentamicin versus ceftriaxone

We found no experimental data directly comparing outcomes following treatment of very severe childhood pneumonia using the recommended antibiotics against ceftriaxone, a common regimen used by clinicians in Kenya. One small trial (n = 97) from Turkey compared benzyl penicillin combined with chloramphenicol and ceftriaxone at day 10 of treatment for radiologically-confirmed severe and very severe pneumonia [17,32] while another small trial (n = 71) compared intravenous benzyl penicillin combined with gentamicin with intravenous amoxicillin-clavulanate in Indian children [17,33] with severe hypoxemic pneumonia. Neither trial reported a superior regimen. Failure to report on the process of randomization and allocation concealment in the study conducted in Turkey was considered a serious limitation and therefore the evidence was graded downwards by one level. This quality of evidence was further downgraded on account of serious indirectness of population and comparison as well as imprecision. The evidence from the study conducted in India was also graded downwards for indirectness of population and comparison and imprecision. The overall quality of evidence was regarded to be very low addressing the question of whether ceftriaxone is better than benzyl penicillin plus gentamicin (Table 6).

**Table 6:** GRADE evidence profile 3: Benzyl penicillin plus gentamicin versus ceftriaxone for very severe pneumonia^†^

Quality assessment					Summary of findings			
					**Number of patients**		**Effect**	**Quality of evidence**

**No of studies/Design**	**Limitations**	**Inconsistency**	**Indirectness**	**Imprecision**	**ceftriaxone**	**benzyl penicillin and gentamicin**	**(P value)**	

*Outcome 1: Time to resolution of signs and symptoms of pneumonia* *Importance: Critical*								

1 RCT [32]	serious^1^	no serious inconsistency	very serious^2^	serious^3^	51	46	(P > 0.05)	Very low

*Outcome 2: Treatment failure/time to resolution of clinical signs of pneumonia* *Importance: Critical*								

1 RCT [33]	no serious limitations	no serious inconsistency	very serious^4^	Serious^5^	33	38	(P > 0.1)	Very low

*Outcome 3: Mortality* *Importance: Critical*								

No studies	-	-	-	-	-	-	-	-

*Outcome 4: Cost* *Importance: Important*								

No studies	-	-	-	-	-	-	-	-

**Overall quality of evidence: Very low quality evidence suggests no advantage of ceftriaxone over benzyl penicillin/ampicillin and gentamicin**								

Benefits or desired effects	Lower risks, less discomfort associated with single injection							

Risks or undesired effects	Potential emergence of extended spectrum beta-lactamase (ESBL)-producing organisms; concern of 'overuse' of important second line drug and what next if it doesn't work?							

Values and preferences	Favourable once-daily dosing schedule							

Costs	Ceftriaxone more costly than penicillin/gentamicin. However availability of cheap generic preparations and additional costs (consumables and human resource) associated with multiple injections may outweigh apparent cost disadvantage of ceftriaxone.							

Feasibility	Ceftriaxone already widely available and in use							

^† ^Clinical question: For HIV-unexposed Kenyan children aged 2 - 59 months without clinical signs of severe malnutrition who meet WHO criteria for very severe pneumonia, should benzyl penicillin/ampicillin plus gentamicin be replaced by ceftriaxone?

^1 ^Limitation due to failure to lack of description of randomisation/allocation concealment

^2 ^Indirectness of comparison (compared penicillin chloramphenicol versus ceftriaxone) and population (Conducted in Turkey),

^3 ^Imprecision due to small sample size

^4 ^Indirectness of: 1) comparison (compared benzyl penicillin gentamicin versus amoxicillin/clavulanate), 2) population (India),

^5 ^Imprecision due to small sample size

#### Grading the evidence

The primary evidence retrieved was from randomized controlled trials and therefore might initially be considered high in quality. However, after subjecting the studies to the GRADE quality assessment process, all the studies were graded downwards leaving no high quality evidence for any of our 5 Kenyan policy questions. Although the GRADE system provides for the inclusion of data from observational studies, we found none that addressed our clinical questions. Our reasons for downgrading evidence, and any challenges with this are highlighted below.

#### Limitations

Limitations that might result in downgrading are reasonably clearly defined in GRADE and include lack of allocation concealment, absence of blinding and large losses to follow up. For pneumonia two trials failed to report allocation concealment [29,32] and blinding was only achieved in the trials comparing cotrimoxazole versus amoxicillin, likely due to practical limitations related to the nature of the interventions in the other trials (e.g. comparisons between injectable versus oral treatments [25,27,28]). Reported losses to follow up were low in all of the identified studies. Despite the global importance of pneumonia as a cause of mortality in children [34] evidence was often inadequate in quantity or quality or both, a finding common to most other topic areas we examined.

#### Inconsistencies

Inconsistency refers to large and unexplained variability in magnitude of effects across studies. Hidden inconsistency becomes more apparent as the number of studies compared increases. With only a few studies available apparent inconsistency was only detected in one instance, in trials comparing cotrimoxazole and amoxicillin for non-severe pneumonia. Straus et al [22] reported superiority of amoxicillin over cotrimoxazole with other studies [23,24] reporting equivalence. Although this trial appeared to influence the conclusions of an influential review suggesting amoxicillin should be the preferred treatment [21] the effect appeared due to the inclusion of a group of children with severe pneumonia. We, therefore, did not downgrade the quality of evidence but instead opted to consider evidence from only those children with non-severe pneumonia, a decision which resulted in three reports indicating equivalence of these drugs in children with only non-severe pneumonia.

#### Indirectness

Indirectness refers to differences between the evidence under review and the clinical question of interest in relation to the PICO elements. We downgraded all the studies reviewed by one level for indirectness of population based on geographic location since they included little or no data from African children. This subjective decision was based on studies suggesting higher risks of treatment failure and mortality in African children with pneumonia [31,35,36]. Interestingly, this position was shared by the Kenyan audience who cited professional experiences to back their distrust of the generalizability of data from Asia. Indirectness was also related to the interventions studied and resulted in downgrading the evidence available for treating very severe pneumonia [32,33], where this, coupled with only data from non-African populations led us to downgrade for indirectness alone by two levels. Amongst other topics we found we also downgraded evidence on the basis that data were relatively old, were available only or predominantly on adults or dealt with outcomes other than those pre-specified as critical.

#### Imprecision

In studies where sample size is small and number of events few, estimated effects are associated with wide confidence intervals and therefore regarded as imprecise. This was relatively common with three trials which recruited less than 100 patients graded downwards by one level for imprecision [29,32,33].

Our ability to conduct meta-analysis incorporating data from all of the studies reviewed was frustrated by differences in reported outcomes [27] or an inability to incorporate data from a large cluster randomized controlled trial [24] (because entry of primary data assumes individual randomization) into a pooled analysis in GRADEpro.

#### Publication Bias

Reliance only on published studies may cause bias if there is a preference for reporting only 'positive' outcomes. Our resources and we suspect those of many in low income settings, precluded detailed searches of grey literature making such a bias likely, particularly where there are already very few published trials. In the case of pneumonia the similarity of studies to those identified in a subsequently published Cochrane review [17] are reassuring but for other topics this remains a threat to our findings.

#### Additional evidence

We did not find any studies reporting on cost-effectiveness of alternative pneumonia treatments. We considered that evidence from observational studies suggesting no clinical difference following treatment of pneumonia in children with beta-lactam antibiotics in the presence of penicillin-resistant or penicillin-sensitive pneumococci [37] to be of potential value when considering recommendations. However, local data on *in vitro *resistance and the clinical effectiveness of antibiotic treatments were either limited or completely lacking respectively.

#### Moving from evidence to recommendations

The GRADE approach indicates that contextual factors including cost, local values and preferences, feasibility, undesired effects and benefits should be taken into account in making a recommendation based on evidence. There is no explicitly defined preferred method for taking these factors into account. Our pragmatic approach was to engage a national guideline development forum attended by a panel of 70 people from academic and policy backgrounds and routine clinical settings. After presenting and discussing the evidence we invited discussion on these contextual considerations (Figure 2) amongst panelists to inform development of recommendations rather than relying on research data alone. However, this forum did not include patient representatives as is recommended in GRADE [38], instead professional health workers were asked to consider this perspective. Such an approach is pragmatic but has clear limitations. However, gaining patients' perspectives, particularly when they are children, is a poorly developed area of practice and research in both low and high income settings alike. How this forum and deliberative process proceeded will be described in detail elsewhere, however here we illustrate how the evidence met with contrasting fates in the process of making recommendations.

Most notable was a strong recommendation against the proposal to adopt oral amoxicillin in place of or even as an alternative to benzyl penicillin/ampicillin for severe pneumonia. This was despite evidence from three large trials suggesting equivalence between the treatments and several additional factors apparently favoring oral amoxicillin including lower cost, more convenient twice daily dosing and reduction in the use of injections in children. In this case the panel essentially discounted moderate quality evidence and felt that such a change in policy should only be informed by locally-generated data. Such an absolute rejection of evidence gathered from trials involving almost 5000 children, something of a surprise to those summarizing it, was also observed for other guideline topics. In this particular case one could speculate that the absence of patient views, children who might receive either multiple injections or oral therapy, might have been an important omission.

Interestingly, strong recommendations were also made against proposals to adopt penicillin plus gentamicin for severe pneumonia and ceftriaxone for very severe pneumonia. Although evidence was largely absent, and thus did not support these proposals, such regimens were admitted to be common local practice. Here a major reason given for strongly rejecting what was already being practiced, in support of existing recommendations, was the perceived need to preserve the alternative treatments as second line regimens in the absence of viable alternatives. It is thus possible that here discussions on the absence of evidence supporting superiority of non-guideline regimens being used in practice may have helped to rebuild confidence in existing guidelines. In line with the evidence presented, the panel made a strong recommendation to abandon chloramphenicol as an alternative treatment to benzyl penicillin/ampicillin and gentamicin for very severe pneumonia. In this case, although the decision was consistent with the moderate quality evidence, much of the data summarized was from non-African children. This contrasts with rejection of similar quality evidence, also from non-African children, referred to above in the case of amoxicillin in severe pneumonia. In this case we speculate that abandoning chloramphenicol may have resonated with existing views that the drug was no longer suitable, having been abandoned by western countries many years ago on the grounds of safety.

## Discussion

We used the GRADE system to review and contextualize the evidence on a number of topics as part of an effort to revise Kenyan national guidelines. We have used the antibiotic treatment of community-acquired pneumonia in Kenyan children to illustrate the approach and some of the challenges encountered. Perhaps one of the most striking findings is the limited availability of high quality evidence generalizable to the population of interest, Kenyan or African children. Whereas it is not feasible for all policies to have supporting data from large randomized controlled trials, we noted a serious shortage of good evidence on a number of other important guideline related questions in pediatric and neonatal care. This contrasts with the situation for a topic such as outpatient malaria, where a recent systematic review managed to include fifty randomized trials [39], reflecting the disparities in interest and funding in child and newborn health.

Where studies are few limitations of the evidence are likely to be greatest. It is hard to detect publication bias, inconsistency is clearly hard to evaluate, imprecision is likely and indirectness related to study site will be common. With low quality data recommendations should generally be weak if the GRADE system is adhered to. We employed a voting system based on an adaptation of the GRADE grid [40] that it was anticipated would allow the consensus group to indicate strong or weak support for a recommendation. However, in almost all cases strong recommendations were made irrespective of the evidence quality. While the decision making process might have influenced this observation we speculate that it may also reflect concern that a weak recommendation does not make for a good national guideline.

Grading the evidence when resources are limited may depend, to a considerable degree, on the subjective decisions of a small number of people. In our case evidence was summarized and graded by only two to three people. In an attempt to engage the local pediatric establishment review teams included people with very limited prior training or experience in this field. While the GRADE approach and software appeared relatively intuitive to use after limited introductory training, decisions on how to grade the quality of evidence, for example down-grading for indirectness, might have influenced subsequent recommendations and inexperience resulting in inappropriate decisions may have been important. The alternative of engaging a team of experienced methodologists in an effort to minimize the potential limitations referred to above would have been associated with substantial increases in the cost of the process challenging the feasibility of this exercise in low income settings [41]. Instead we attempted to minimize the potential for error and bias through adopting measures such as having two authors independently generate GRADE evidence profiles and facilitating a transparent reporting of the reviews.

The GRADE approach promotes a more transparent contextualization of evidence to produce final recommendations. The degree to which it is reasonable to 'abandon' evidence in favor of context is perhaps debatable. It appeared to us that moderate quality evidence was on occasions influential and on others ignored. Thus in the absence of likely significant additional contextual factors (such as costs or feasibility) decisions appeared to be based largely on the preferences of the people assembled and their views on patient preferences. The dynamics of the group process, the choice of the discussion moderator and his interaction with the recommendations panel, the composition of this panel, the mode of presentation of the evidence and other factors may all have contributed to the direction and strength of final recommendations [42-44]. We will report observations on these potentially important aspects of the use of GRADE in real life settings in full in due course.

Although our application of the GRADE system may have implications for the validity of our final recommendations, it can be argued that our approach was in fact an improvement on previous exercises in national guideline development that lacked transparency [45]. Previously, the local process involved the adaptation of recommendations issued by the WHO through a relatively closed process by a small panel of local experts [41]. The weaknesses of this practice were compounded further by flaws in the process guideline development at the WHO [46]. As more groups in low income settings use GRADE open sharing of resources and experiences is likely to gain importance, hence all the material we generated in this exercise is freely available online [18]. Such sharing of reviews is important to avoid duplication of efforts and should help set priorities for undertaking and disseminating formal systematic reviews. Sharing lessons from users of GRADE will also be valuable for the further refinement and adaptation of this system to optimize guideline development in settings where the capacity is limited and locally generated evidence lacking.

## Competing interests

The authors declare that they have no competing interests.

## Authors' contributions

AA supported by ME and NO was primarily responsible for the systematic review of pneumonia case management. AA produced the first draft of the report on use of GRADE that was further developed with input from ME and NO. All authors reviewed and approved the final version of the manuscript.

## Pre-publication history

The pre-publication history for this paper can be accessed here:

http://www.biomedcentral.com/1471-2431/12/1/prepub
